# It’s a small world after all

**DOI:** 10.1007/s12471-012-0291-9

**Published:** 2012-05-25

**Authors:** M. J. de Boer, H. Suryapranata

**Affiliations:** University Medical Center St Radboud, Geert Grooteplein 10, 6525 GA Nijmegen, the Netherlands

The terminology of first, second and third world dates from the times of the cold war and refers to the United States of America and its allies, the old communistic countries including China, and the non-aligned countries (among them Indonesia), respectively. As the world moves on in the 21st century, rapid changes in this concept can be observed. In the current issue of our journal the title of the article by Dharma et al., is somewhat misleading as “the third world” as we knew it does not exist anymore [1]. They describe their experience with treatment of ST-elevation myocardial infarction (STEMI) in the city of Jakarta, Indonesia.

The Indonesian health care providers represent a mix of public and private institutions, roughly half of them owned by private investors [2]. Experience, logistics and patient characteristics may differ profoundly in different parts of Indonesia, but it was demonstrated that primary percutaneous coronary intervention (pPCI) for STEMI can obtain good results with regard to restoration of TIMI-3 flow in the infarct-related vessel, in a “third world” setting [2, 3]. However, time delays between symptom onset as well as between admission and first balloon inflation are considerably longer [3]. The main problem with pPCI is that it is not readily available to most patients with STEMI. Many STEMI patients still receive thrombolytic therapy or no reperfusion at all, owing to the limited availability of pPCI and other resources. Besides, many community hospitals do not have the annual PCI volume needed to maintain the skills of the interventionalist and the cardiac catheterization laboratory staff, which is crucial in the treatment of STEMI patients (especially when they are at high-risk or hemodynamically unstable.) As the authors correctly state, the percentage of STEMI patients not receiving reperfusion therapy is too high - almost 60 % - and this number needs to be reduced bij programmes [4]. Furthermore, of the two reperfusion options, pPCI is by far the best treatment and should be available for all who need it, whether it is in the “first” or in the “third world”. For advanced medical care, Indonesia relied and was dependent untill very recently, on hospitals in other countries like Singapore. They provided heart surgery and coronary interventions for many people from Indonesia over the years, but in the case of STEMI patients there is no time for transportation and any delay is deleterious, creating the need for well equipped cardiac intervention centers in Indonesia, like the National Cardiovascular Center “Harapan Kita” in Jakarta.

But many questions remain: What about the use of adjunctive but expensive therapy, like glycoprotein 2b/3a blockers, new drug eluting stents and intra-aortic balloon pumping in a “third world” setting? Many patients will need implantable defibrillator and/or resynchronization treatment as well, after surviving a STEMI. Dharma and co-workers do not give information on this but fibrinolytic therapy remains one of the cornerstones of therapy in Indonesia, especially in isolated and rural areas, but also cities like Jakarta. The city of Jakarta itself - some 10 million people, and by far the largest city in South-East Asia - is suffering from a large traffic and transportation infarct and timely arrival in a hospital with interventional cardiology facilities (which is crucial for a good clinical outcome) may simply be impossible.

But the main problem remains unaddressed: how to provide general access to optimal medical care for STEMI in “third world” countries? (By the way, what about the 50 million of uninsured citizens in the U.S.A. - supposed to be part of the “first world” - where paradoxically the highest costs of medical care per person are reported [5].)

**Fig. 1:**
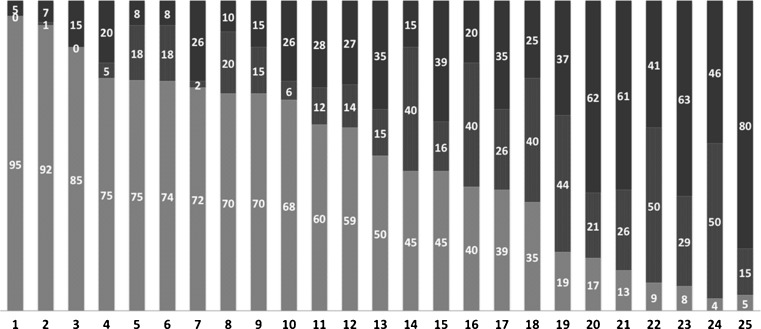
STEMI treatment in European hospitals in 2010 (data from national registries or surveys). 100 % of all hospitalized STEMI patients in each given country; light grey, STEMI patients treated by primary PCI; dark grey: STEMI patients treated by thrombolysis; black, STEMI patients not treated with any reperfusion. Countries: 1: the Netherlands; 2: Czech Republic; 3: Slovenia; 4: Norway; 5: Denmark, 6: Belgium; 7: Poland; 8: Switzerland; 9: Croatia; 10: Sweden; 11: Hungary; 12: Germany; 13: Israel; 14: Finland; 15: Italy; 16: Spain; 17: Slovakia; 18: Austria; 19: Portugal; 20: Serbia; 21: Bulgaria; 22: Greece; 23: Turkey; 24: Romania; 25: Russia. (Provided very kindly by professor Petr Widimsky)

And how about pPCI facilities in Europe (the “old continent” or “first world”)? Although pPCI has been widely implemented, there are still major differences in PCI facilities and reperfusion strategy among European countries. In fact, too many STEMI patients still do not receive any reperfusion therapy in some European countries (Fig. 1). But more importantly, many European countries have made tremendous progress in pPCI. In fact, implementation of a pPCI strategy, relying on better logistics and hospital infrastructure has increased the overall use of reperfusion therapy, resulting in a significant decrease in cardiovascular mortality. However, it took more than 15-years since the first publications in 1993 to achieve this stage. Therefore, nationwide implementation of pPCI remains to be developed, not only in the “third world”, but also in many European countries. It was recently estimated that worldwide, more than 10.000 hospitals provide invasive reperfusion therapy in patients with STEMI nowadays. Still there is a continuing controversy about the acceptable time-window for pPCI. The evidence supports an acceptable pPCI-related delay of 80–120 min [6], and the median door to treatment time of 38 and 95 min for “needle” and “balloon” respectively, reported from Jakarta “Harapan Kita” National Hospital stands well against other countries.

Indonesia seems to be on the right path: large companies and the government already installed health care systems for open access and the formerly “elitary treatment” only for those who are very rich or well to do is abandonned but not yet history. Dharma and colleagues are to be congratulated on this first step by sharing their data with us.
